# The Effects of Plant Virus Infection on Polarization Reflection from Leaves

**DOI:** 10.1371/journal.pone.0152836

**Published:** 2016-04-21

**Authors:** Daniel J. Maxwell, Julian C. Partridge, Nicholas W. Roberts, Neil Boonham, Gary D. Foster

**Affiliations:** 1 School of Biological Sciences, University of Bristol, 24 Tyndall Avenue, BS8 1TQ, United Kingdom; 2 School of Animal Biology and Oceans Institute, University of Western Australia, Crawley, Perth, Western Australia 6009; 3 The Food and Environment Research Agency, Sand Hutton, York, YO41 1LZ, United Kingdom; University of the West of England, UNITED KINGDOM

## Abstract

Alteration of leaf surface phenotypes due to virus infection has the potential to affect the likelihood of colonisation by insect vectors, or to affect their feeding activities. The aim of this study was to investigate whether viruses that rely on insects for their transmission, and which can be sensitive to the polarization of light, affect the percentage polarization of light reflected from leaves. We also set out to discover whether a correlation exists between the expression of *ECERIFERUM* (*CER*) genes involved in cuticular wax synthesis and the polarization of the light reflected from the leaf surfaces. It was found that the aphid-vectored viruses *Potato virus Y* and *Cucumber mosaic virus* (CMV) caused significant reductions in the percentage polarization of light reflected from the abaxial surfaces of leaves of *Nicotiana tabacum*, whereas the non-insect-vectored viruses *Tobacco mosaic virus* and *Pepino mosaic virus* did not induce this effect. In *Arabidopsis thaliana*, there was little difference in the impacts of CMV and the non-insect-vectored *Turnip vein clearing virus* on polarization reflection, with both viruses increasing the percentage polarization of light reflected from the abaxial surfaces of leaves. There was a trend towards increased accumulation of *CER6* transcripts in *N*. *tabacum* and *A*. *thaliana* when infected with aphid-vectored viruses. No significant effect of infection on trichome densities was found in *A*. *thaliana*, suggesting that alterations to the formation of cuticular waxes may be the more likely phenotypic change on the leaf surface contributing to the changes in polarization reflection. The possible impacts and adaptive significance of these effects with regard to viral transmission by insects are discussed.

## Introduction

The majority of vector-transmitted plant viruses are spread between host plants by insects, such as aphids, whiteflies and thrips [1]. In an adaptive context viruses are not passive players during transmission, and have been shown to alter plant and vector interactions in ways that enhance viral transmission strategies [2–8]. A review of the literature by Mauck *et al*. (2012) found that the effects of plant viruses on vector behaviour, mediated through the impacts of infection on host plant phenotypes, are generally conducive to transmission [2]. In both persistently transmitted (PT) (with the virus remaining infective on the vector from several hours to days, and requiring sustained aphid feeding for acquisition and inoculation on to plants) and non-persistently transmitted (NPT) (where the virus is only retained briefly on the insect and is acquired or inoculated during brief probing of the plant epidermal cells by aphids) [1,9] viruses function to increase the initial attractiveness of infected plants to vectors [2], although a small number of studies report preferential attraction of aphids to healthy plants [10,11].

Plant viruses can affect the attraction of insect vectors through modified olfactory signals [3–5,12,13]. Changes to the nutritional quality of infected plants are also thought to affect aphid feeding and settlement [4,14,15] and differences in vector feeding behaviours on virus-infected plants in comparison to on healthy plants have been reported [5,6,16]. Changes to the structure of leaf surfaces have received little attention in comparison and virus-induced alterations to the leaf surface could affect the tactile perception of a leaf by insects. Changes to the cuticular waxes can affect the recognition of host plants by aphids [17] and leaf hairs (trichomes) can interfere with insect movement and settlement [18]. Additionally, the visual appearance of the leaf could be altered, as surface features such as cuticular waxes and trichomes are a key determinant of reflectance properties; in particular the reflection of polarized light [19–21]. Whether plant viruses alter leaf surface phenotypes in ways which could influence polarization reflection has never been addressed.

Sensitivity to the polarization of light is a common visual ability across the animal kingdom [22]. Many species of insect have polarization-sensitive visual systems [23] and use the polarization pattern of the sky for navigation (e.g. bees [24], ants [25,26] and dung beetles [27]), the polarization cue from water for locating habitats (e.g. dragonflies [28]),polarization signals for flower identification (in bees [29]) and possibly discrimination of egg-laying and feeding sites (in butterflies [30,31]). To our knowledge, polarization sensitivity has never been investigated in the common hemipteran insects which act as plant viral vectors (e.g. aphids and whiteflies), but the few studies that have investigated the architectures of the aphid eyes indicate that they possess a typical apposition compound eye with a fused rhabdomeric photoreceptors and the interesting inclusion of a separate structure called the triommatidium that contains only three facets and is situated on lateral tubercles [32]. Therefore if, like many other insects with apposition type eyes that possess polarization sensitivity (see above) the viral vectors are polarization sensitive, then it is a plausible hypothesis that virus-induced alterations to polarization reflections from leaves could affect the visual attractiveness of infected plants.

The leaves of plants have the ability to polarize reflected light across a range of viewing angles for obliquely incident light and any reflections are predominately linearly polarized at an angle parallel to the plane of the leaf surface [33]. The angle of polarization refers to the predominant angle of oscillation of light’s electric field vector, whilst the degree of polarization, often expressed as a percentage polarization, denotes the extent to which the light is polarized; in fully linearly polarized light the wave’s oscillation is confined to one plane, whereas in partially polarized light there is distribution in angles of individual waves, about a measurable mean [21]. When light is incident at Brewster’s angle (around 55 degrees from the vertical for a leaf in air) onto a smooth surface, the reflected light can approach being 100% polarized. However, the surface roughness is a key determinant of the polarizing properties of the leaf surface; smoother surfaces reflect with a higher percentage polarization than rougher surfaces [19,21]. As such, the structure of the leaf, for example its microtopology, greatly influences the polarization of any reflections [19,21]. In contrast, light reflected from the leaf interior undergoes multiple scattering and has a much lower percentage polarization than the surface-reflected light [33]. The net percentage polarization reflection from a leaf results from the relative contributions of surface-derived and internally-derived reflectance [21], as well as the angle of view and illumination.

Epicuticular waxes, which comprise the outermost layer of the leaf cuticle, are an important determinant of polarization reflection properties [21] and changes to these provide a possible mechanistic pathway by which viruses can alter a polarization dimension to a visual signal. The synthesis of alkanes, aldehydes, secondary alcohols, ketones, primary alcohols and wax esters, which make up the epicuticular waxes, and are derived from very long chain fatty acids (VLCFAs), occurs in the epidermal cells [34]. In *Arabidopsis thaliana*, the best characterised genes involved in wax synthesis are the *ECERIFERUM* (*CER*) genes, including *CER6*, which encodes a condensing enzyme that catalyses the extension of VLCFA chains by two carbon increments [35]; *CER5*, an ABC transporter involved in the movement of waxes across cell membranes [36]; *CER8*, which facilitates the extension of fatty acids through the addition of CoA [37]; *CER3*, which catalyses the conversion of long chain fatty acyl-CoAs to alkanes [38]; *CER7*, a positive regulator of *CER3* [39]; and *CER9*, a ubiquitin ligase which is thought to negatively regulate leaf wax accumulation [40]. If viruses that use insects as a vector are able to adaptively alter the visual signals broadcast by infected plants, then we would hypothesize expression levels of *CER* genes would be altered to modify the epicuticular waxes and therefore change the reflective polarization properties of the infected host leaves. Moreover, viruses that do not use insects as a vector should not orchestrate the same changes.

In this work, we aimed to test these suggested hypotheses. We measured the percentage polarization of light reflected from leaves of *Nicotiana tabacum* infected with *Potato virus Y* (PVY) and *Cucumber mosaic virus* (CMV) (aphid-borne viruses) and *Tobacco mosaic virus* (TMV) and *Pepino mosaic virus* (PepMV) (non-insect-transmitted viruses) respectively. Furthermore, we analysed levels of expression of several *CER* genes involved in cuticular wax synthesis. Similar approaches were carried out in *A*. *thaliana*, using the non-insect-transmitted *Turnip vein clearing virus* (TVCV) and aphid-borne CMV. Finally, the effects of viral infection on trichome densities were also compared between healthy and infected *A*. *thaliana* leaves to indicate whether variation in trichome numbers may also influence any alterations to polarization of the reflection.

## Results

### Effects of viral infection on polarization reflection from *N*. *tabacum* leaves

**Fig 1:**
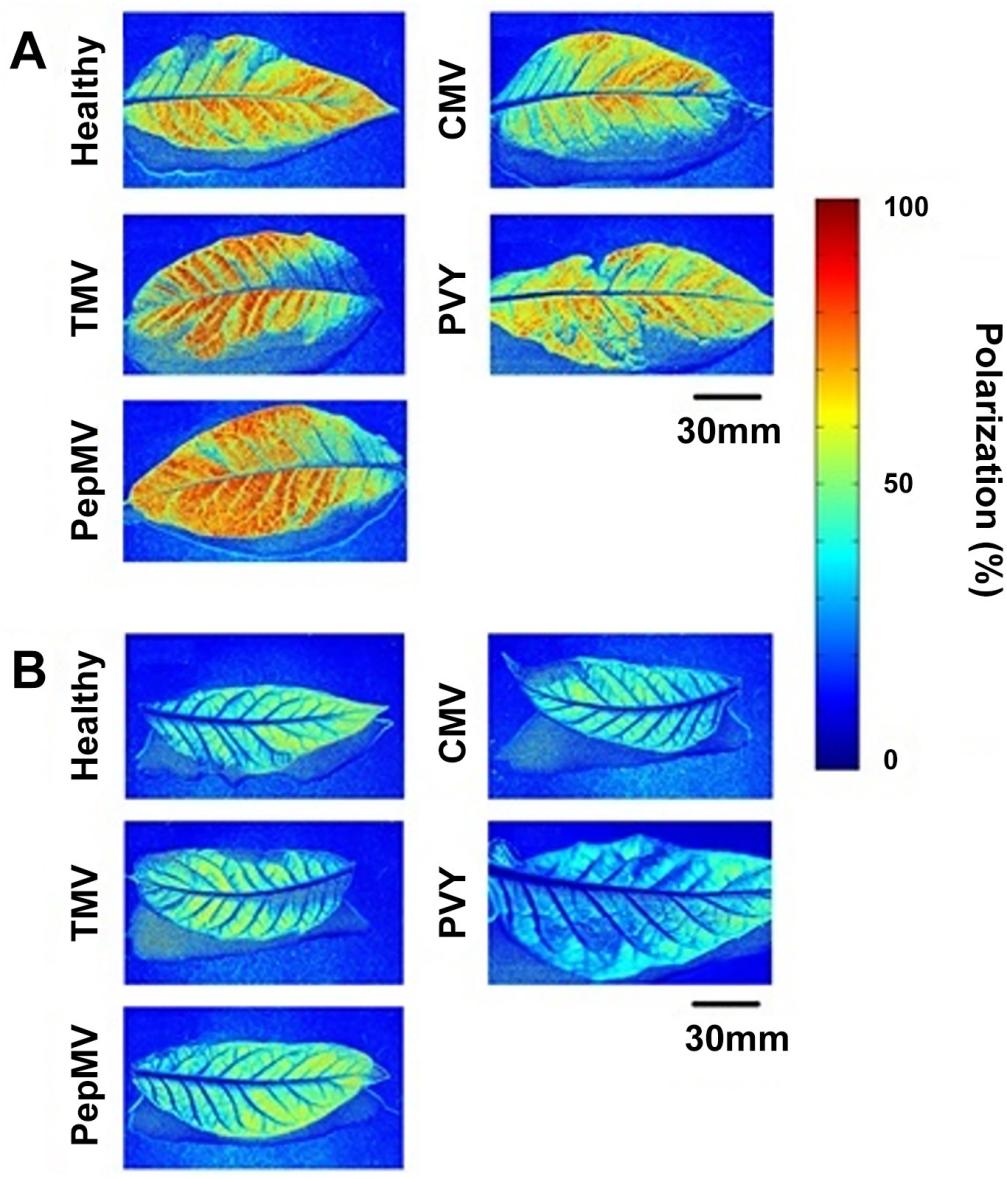
Example false colour images showing the percentage polarization of blue channel light reflected from healthy, TMV, PepMV, PVY or CMV-infected leaves of *N*. *tabacum* on the adaxial (A) or abaxial (B) surfaces at 21 days post inoculation (DPI). The percentage polarization at each pixel is represented by colour, as shown in the scale bar.

The average percentage polarization of light reflected from the adaxial and abaxial surfaces of *N*. *tabacum* leaves infected with the non-insect-vectored viruses *Tobacco mosaic virus* (TMV) and *Pepino mosaic virus* (PepMV), or the aphid-vectored viruses PVY and CMV, was analysed by polarization imaging (Fig 1).

### TMV and PepMV infections (non-insect-vectored viruses)

**Fig 2:**
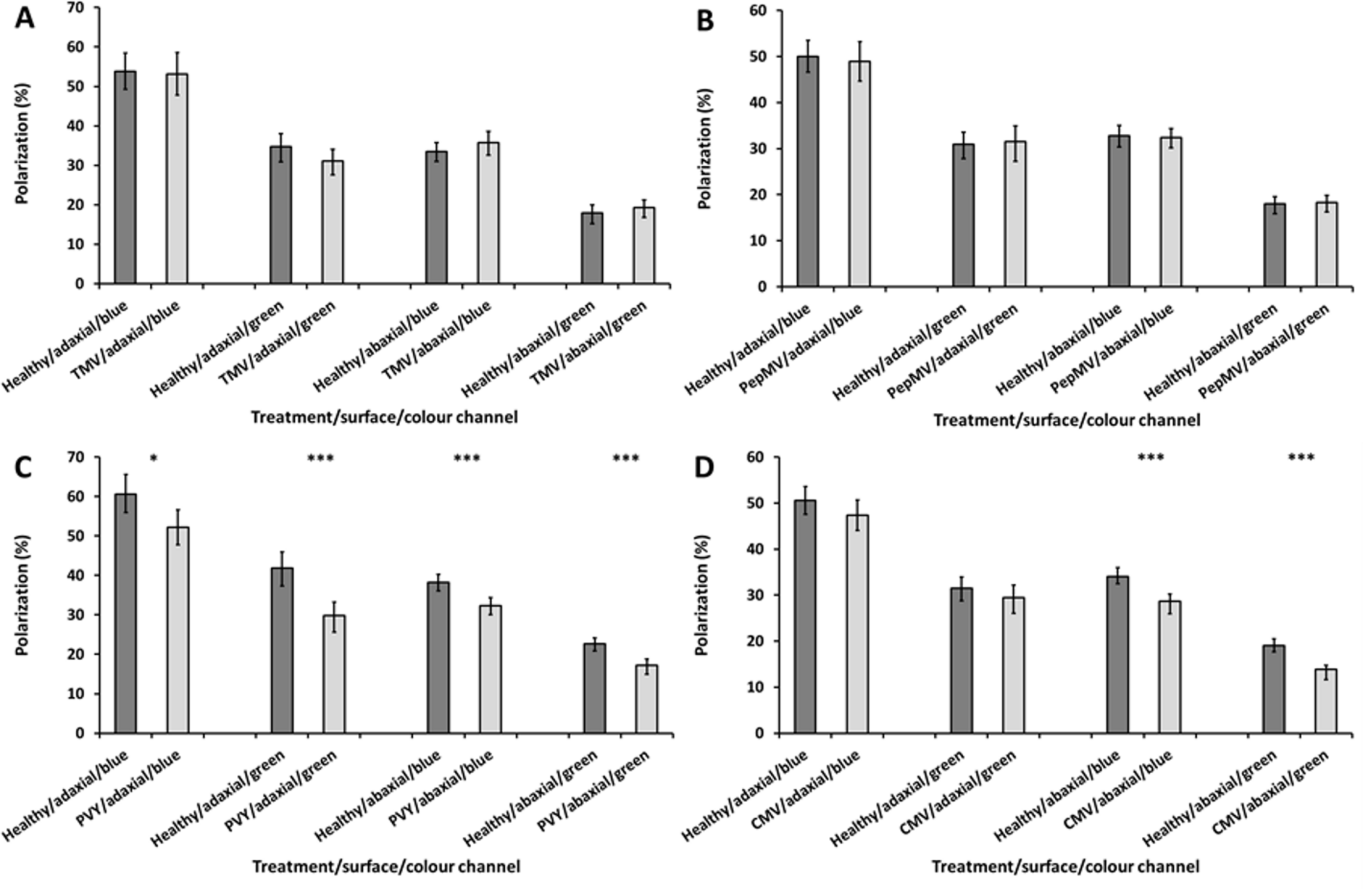
Average percentage polarization of light reflected from the adaxial and abaxial surfaces of TMV (A), PepMV (B), PVY (C) and CMV (D) infected *N*. *tabacum* leaves in comparison to healthy control leaves, in blue and green channel light. Error bars denote 95% confidence intervals; asterisks indicate statistically significant differences between healthy and infected leaves (*<0.05; ***<0.001). A total of 27–33 leaves (with each leaf removed from a separate plant) were analysed within each of the four viral treatments, and were compared to similar numbers of healthy leaves. All imaging experiments were performed at 21 DPI.

TMV infection had no significant impact on the percentage polarization of light reflected from the adaxial or abaxial surfaces of *N*. *tabacum* leaves (in the blue channel a decrease of 0.79%, t-test: t = 0.195, df = 52, *P* = 0.846; in the green channel a decrease of 2.61%, t-test: t = 1.54, df = 52, *P* = 0.13) (Fig 2A).

Similarly, TMV infection did not significantly affect the percentage polarization of light reflected from the abaxial surfaces (in the blue channel an increase of 2.07%, t-test: t = -1.181, df = 54, *P* = 0.243; in the green channel an increase of 1.3%, t = -0.814, df = 54, *P* = 0.375) (Fig 2A).

The other non-insect-vectored virus, PepMV, also had no significant effect on the percentage polarization of light reflected from the leaves (Fig 2B). Adaxially, the percentage polarization of light reflected from PepMV-infected leaves was not significantly different to that reflected from healthy leaves (in the blue channel a decrease of 1.06%, t-test: t = 0.314, df = 52, *P* = 0.755; in the green channel an increase of 0.58%, t = 0.58, df = 52, *P* = 0.723) (Fig 2B). Similarly on the abaxial surfaces, the reflected polarization from the infected leaves was not significantly different to healthy leaves (in the blue channel a reduction of 1.06%, t-test: t = 0.285, df = 60, *P* = 0.777; in the green channel an increase of 0.58%; t-test, t = -0.244, df = 60, *P* = 0.808) (Fig 2B).

### PVY and CMV infections (aphid-vectored viruses)

In contrast, the aphid-vectored viruses PVY and CMV both had significant impacts on the percentage polarization of light reflected from *N*. *tabacum* leaves.

On the adaxial surfaces, PVY-infected leaves were less polarizing than healthy leaves, by 8.34% and 12.07% in the blue and green channels respectively (independent samples t-test: blue, t = 2.688, df = 54, *P* = 0.01; green, t = 4.36, df = 54, *P*<0.001) (Fig 2C).

Similar decreases in the percentage polarization were also observed from the abaxial surfaces of PVY infected leaves; by 5.94% and 5.47% in the blue and green channels respectively (independent samples t-test: blue, t = 4.09; df = 56, *P*<0.001; green, t = 4.557, df = 56, *P*<0.001) (Fig 2C).

On CMV-infected leaves the percentage polarization of light reflected from the adaxial surface was lower than for healthy leaves, by 3.18% and 2.09% in the blue and green channels respectively (Fig 2D), although these are not statistically significant differences (Mann Whitney test: blue, z = -1.176, n = 62, *P* = 0.24; green, z = -0.936, n = 62, *P* = 0.349).

However, the abaxial surfaces of CMV-infected leaves were less polarizing than healthy leaves, by 5.94% and 5.47% in the blue and green channels respectively (Fig 2D) (independent samples t-test: blue, t = 4.012, df = 64, *P*<0.001; green, t = 5.1, df = 64, *P*<0.001)

### Effects of viral infection on expression of wax synthesis genes in *N*. *tabacum*

Due to the influence of leaf surface features on polarization reflection, the effects of TMV, PepMV, PVY and CMV infections on the expression levels of genes involved in cuticular wax synthesis were analysed in *N*. *tabacum*. The levels of expression of *CER3*, *CER5* and *CER6* transcripts were investigated in leaves systemically infected with TMV, PepMV, PVY or CMV.

### TMV and PepMV infections (non-insect-vectored viruses)

**Fig 3:**
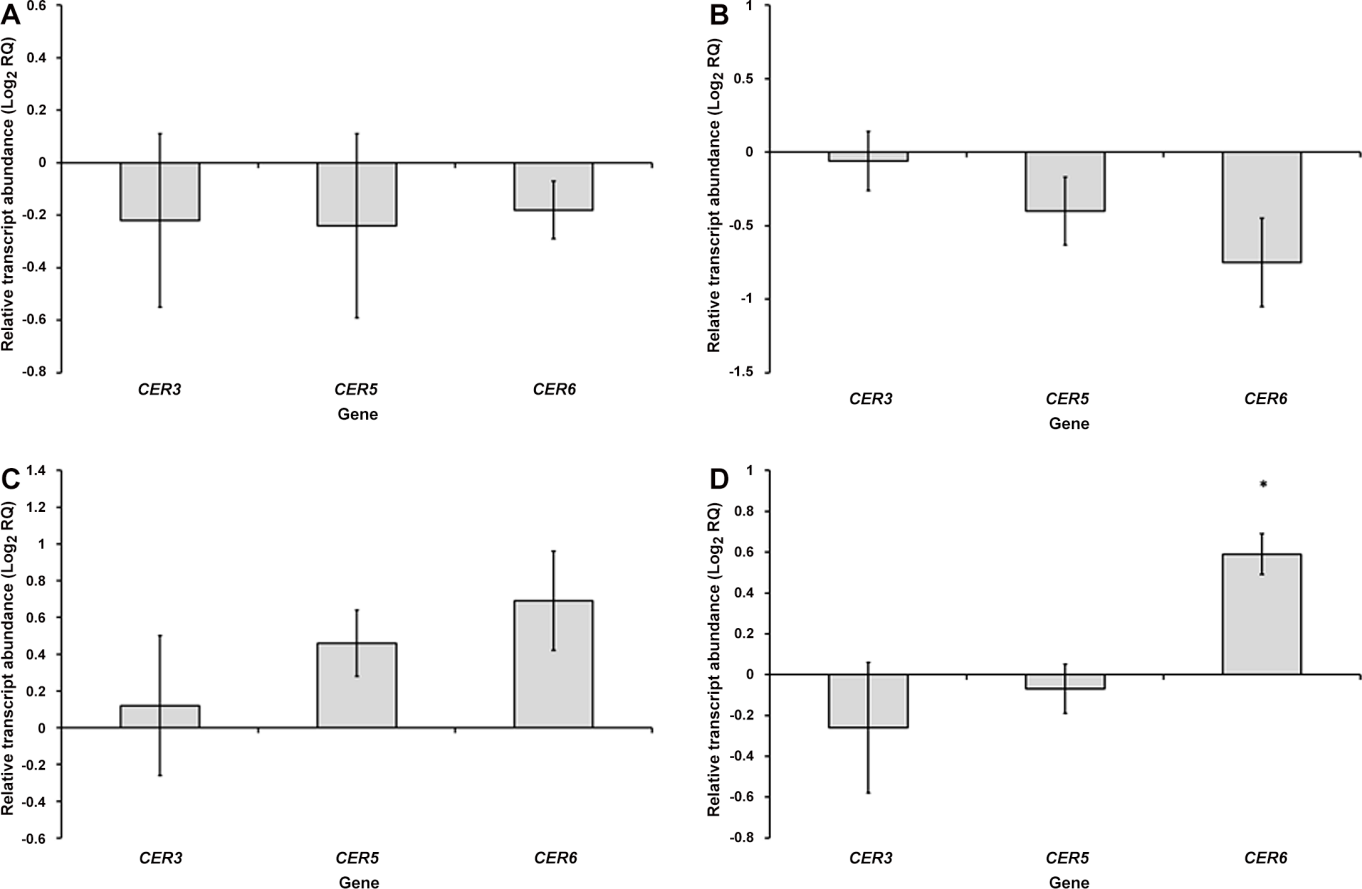
Transcript levels of *CER3*, *CER5* and *CER6* in *N*. *tabacum* leaves systemically infected with TMV (A), PepMV (B), PVY (C) or CMV (D) at 7 DPI, expressed relative to transcript levels in healthy leaves (as Log_2_ RQ). Error bars for each gene denote the standard error of the mean relative change in expression between healthy and infected samples (across the three biological replicates), asterisks indicate statistically significant differences between healthy and infected leaves (*<0.05).

In leaves infected with TMV, *CER3*, *CER5* and *CER6* showed non-significant reductions in transcript levels relative to healthy leaves (one sample t-test: *CER3*, t = -0.694, df = 3, *P* = 0.538; *CER5*, t = -0.769, df = 3, *P* = 0.546; *CER6*, t = -1.603, df = 3, *P* = 0.207) (Fig 3A).

Similar results were obtained in leaves infected with PepMV (one sample t-test t-test: *CER3*, t = -0.296, df = 2, *P* = 0.795; *CER5*, t = -1.767, df = 2, *P* = 0.219; *CER6*, t = -2.522, df = 2, *P* = 0.128) (Fig 3B).

### PVY and CMV infections (aphid-vectored viruses)

Leaves infected with PVY displayed non-significant increases in the abundances of *CER3*, *CER5* and *CER6* transcripts (one sample t-test: *CER3*, t = 0.308, df = 2, *P* = 0.787; *CER5*, t = 2.51, df = 2, *P* = 0.129; *CER6*, t = 2.549, df = 2, *P* = 0.126) (Fig 3C).

CMV infection caused *CER6* to be significantly upregulated, by 1.5 times relative to uninfected leaves (one sample t-test: t = 5.584, df = 2, *P* = 0.031). There was little impact of CMV infection on the expression levels of *CER3* or *CER5* (one sample t-test: *CER3*, t = -0.833, df = 2, *P* = 0.493; *CER5*, t = -0.577, df = 2, *P* = 0.622) (Fig 3D).

### Effects of viral infection on polarization reflection from *A*. *thaliana* leaves

**Fig 4:**
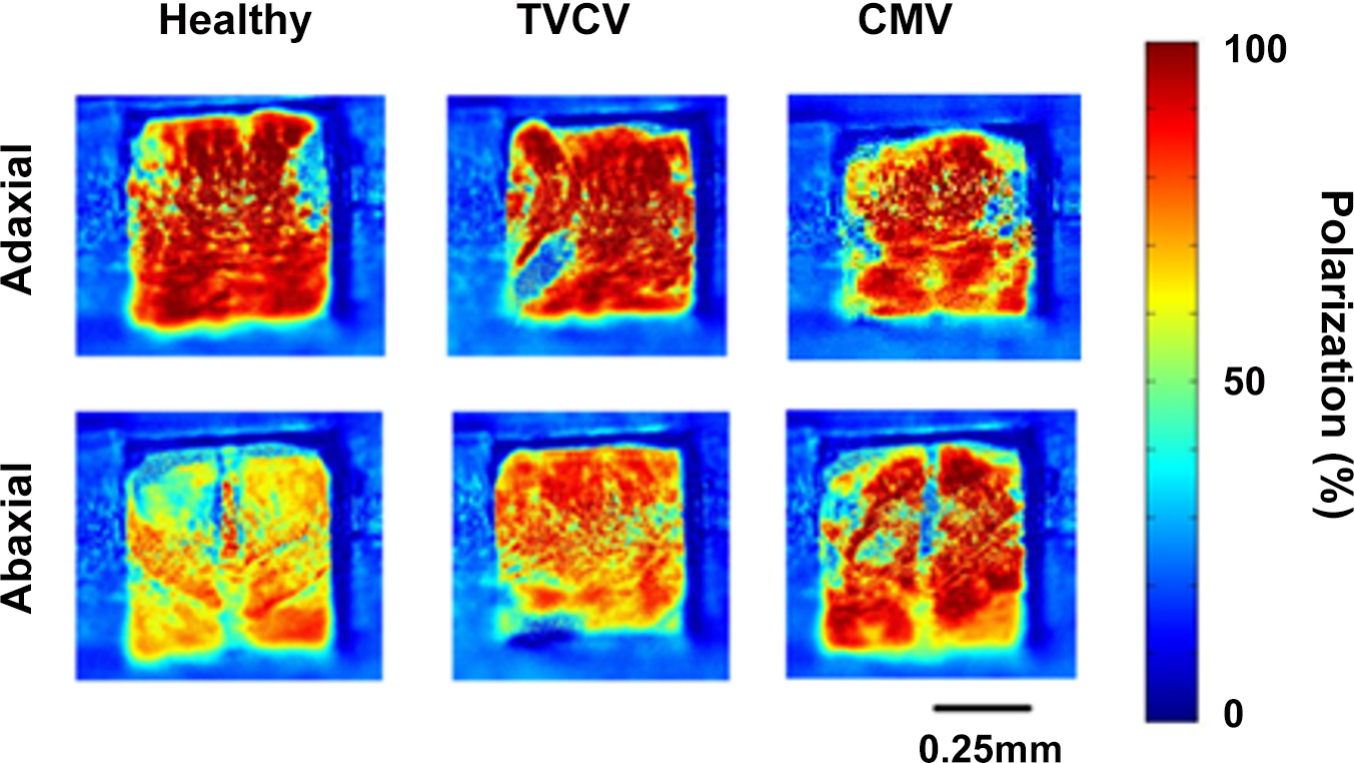
Example false colour images showing the percentage polarization of light reflected from healthy, TVCV or CMV-infected leaves of *A*. *thaliana* on the adaxial or abaxial surfaces at 21 days post inoculation (DPI). The percentage polarization at each pixel is represented by colour, as shown in the scale bar.

Following the investigations in *N*. *tabacum*, similar experiments were performed in *A*. *thaliana*. CMV was inoculated onto this host as an example of an aphid-vectored virus, whilst TVCV, a *Tobamovirus*, was used as an example of a non-insect-vectored virus (Fig 4 for example images).

### TVCV infection (a non-insect-vectored virus)

**Fig 5:**
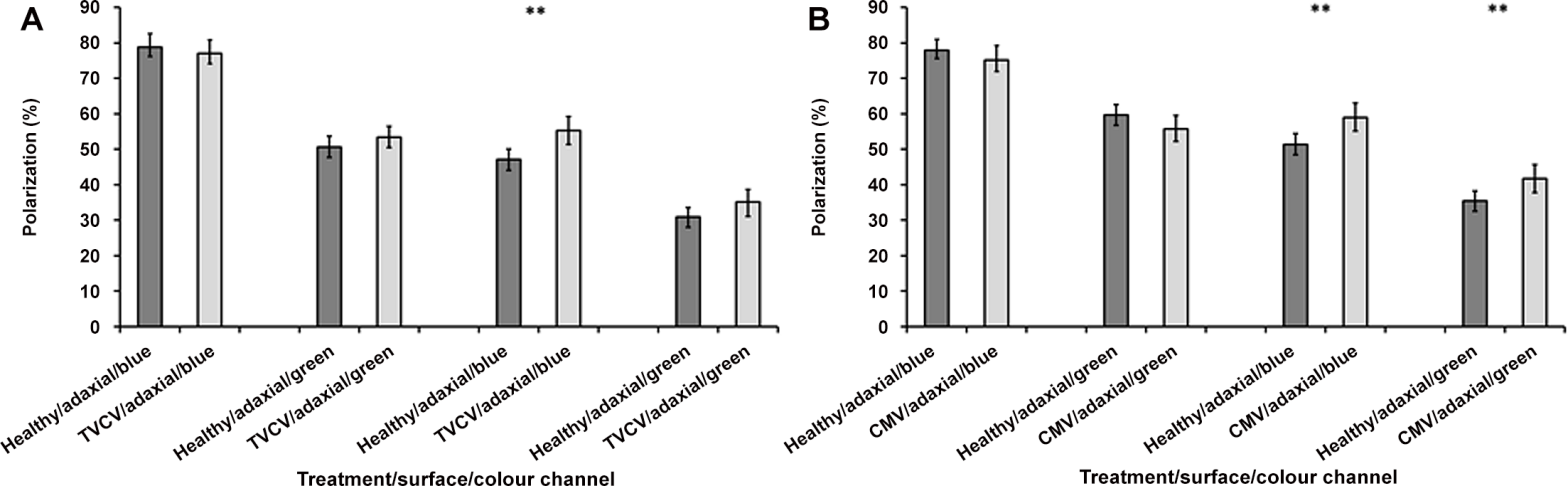
Average percentage polarization of light reflected from the adaxial and abaxial surfaces of TVCV (A) or CMV (B) infected *A*. *thaliana* leaves, in comparison to healthy controls, in the blue and green channels. Error bars denote 95% confidence intervals; asterisks indicate statistically significant differences between healthy and infected leaves (** P<0.01). A total of 33 TVCV-infected leaves and 24 CMV-infected leaves (from separate plants) were analysed and compared to similar numbers of healthy leaves. All imaging experiments were performed at 21 DPI on systemically infected rosette leaves.

On the adaxial surface, the percentage polarization of the reflections from TVCV-infected leaves were not significantly different to those from healthy leaves, in the blue or green channels (independent samples t-test: blue, t = 0.937, df = 64, *P* = 0.352; green, t = -1.232, df = 64, *P* = 0.223) (Fig 5A).

In contrast, on the abaxial surfaces the infected leaves polarized the reflections significantly more in the blue channel (with an 8.17% increase, t-test: t = -3.32, df = 64, *P* = 0.001). An increase, although not significantly different, in the percentage polarization was also seen in the green channel (a 4.22% increase, t-test: t = -1.927, df = 64, *P* = 0.058) (Fig 5A).

### CMV infection (an aphid-vectored virus)

The percentage polarization of light reflected from the adaxial sides of healthy or CMV-infected leaves was not significantly different (independent samples t-test: blue, t = 1.132, df = 46, *P* = 0.264; green, t = 1.621, df = 46, *P* = 0.112) (Fig 5B).

However, from the abaxial surfaces there were increases of 7.55% and 6.33% in the percentage polarization reflection from CMV-infected leaves, in the blue and green channels respectively (Mann Whitney test: blue, z = -3.114, n = 48, *P* = 0.002; green, z = -3.361, n = 48, *P* = 0.001) (Fig 5B).

### Effects of viral infection on expression of wax synthesis genes in *A*. *thaliana*

Following on from the similar analysis above, the accumulation of transcripts of the *CER3*, *CER5*, *CER6*, *CER7*, *CER8* and *CER9* wax synthesis genes in *A*. *thaliana* leaves was investigated by qPCR, in rosette leaves systemically infected with TVCV or CMV.

### TVCV infection (a non-insect-vectored virus)

**Fig 6:**
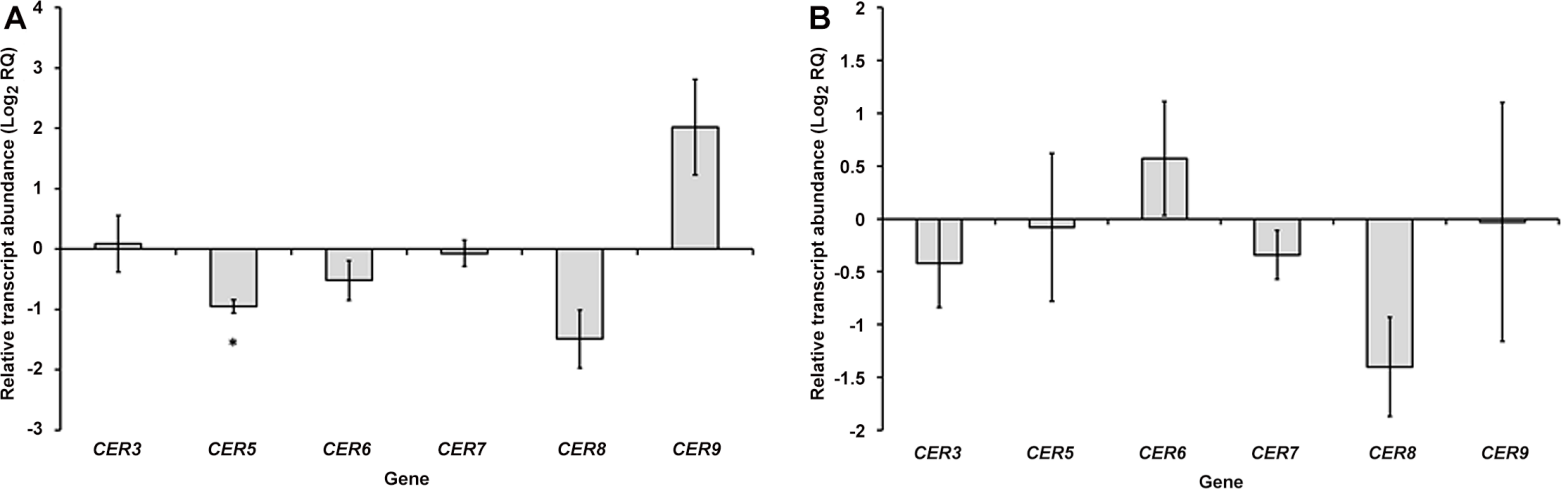
Transcript levels of *CER3*, *CER5*, *CER6*, *CER7*, *CER8* and *CER9* in *A*. *thaliana* leaves systemically infected with TVCV (A) or CMV (B) at 14 DPI, expressed relative to transcript levels in healthy leaves (as Log_2_ RQ). Error bars for each gene denote the standard error of the mean relative change in expression between healthy and infected samples (across the three biological replicates), asterisks indicate statistically significant differences between healthy and infected leaves (*<0.05).

In TVCV-infected leaves, *CER5* was significantly downregulated to around half of the level observed in healthy leaves (one sample t-test: t = -9.046, df = 2, *P* = 0.012). The *CER8* gene also displayed a marked reduction in transcript abundance, to 0.36 times the level in healthy samples, although this was not a statistically significant reduction (one sample t-test: t = -3.101, df = 2, *P* = 0.09). There was little effect of TVCV infection on *CER3* or *CER7* expression (one sample t-test: *CER3*, t = 0.191, df = 2, *P* = 0.866; *CER7*, t = -0.296, df = 2, *P* = 0.8), whilst *CER6* transcripts showed a non-significant reduction in accumulation. *CER9* transcript levels showed a very large variation between replicates, with a non-significant four-fold increase on average (one sample t-test: t = 2.565, df = 2, *P* = 0.124) (Fig 6A).

### CMV infection (an aphid-vectored virus)

In CMV-infected leaves, the *CER6* gene showed an average upregulation in expression by around 1.5 times, although this is not a statistically significant increase (one sample t-test: t = 1.059, df = 2, *P* = 0.401). *CER5* showed very little change in transcript accumulation following infection (one sample t-test: t = -0.12, df = 2, *P* = 0.92), and there was also little effect of CMV infection on the accumulation of *CER3* or *CER7* transcripts (one sample t-test: *CER3*, t = -1.006, df = 2, *P* = 0.42; *CER7*, t = -1.741, df = 2, *P* = 0.279). CMV infection led to a non-significant reduction in *CER8* expression, to around 40% of the level observed in healthy leaves (one sample t-test: t = -3.008, df = 2, *P* = 0.095). There was a high variation in *CER9* transcript levels; with the average level of *CER9* transcript being very similar in CMV-infected leaves to in uninfected leaves (one sample t-test: t = -0.026, df = 2, *P* = 0.98) (Fig 6B).

### Trichome densities on TVCV and CMV-infected *A*. *thaliana*

In addition to changes in wax composition, the percentage polarization reflection from leaves could also be influenced by the number of trichomes present on the surface. Therefore, the densities of trichomes were compared between healthy and systemically TVCV or CMV-infected rosette leaves.

**Fig 7:**
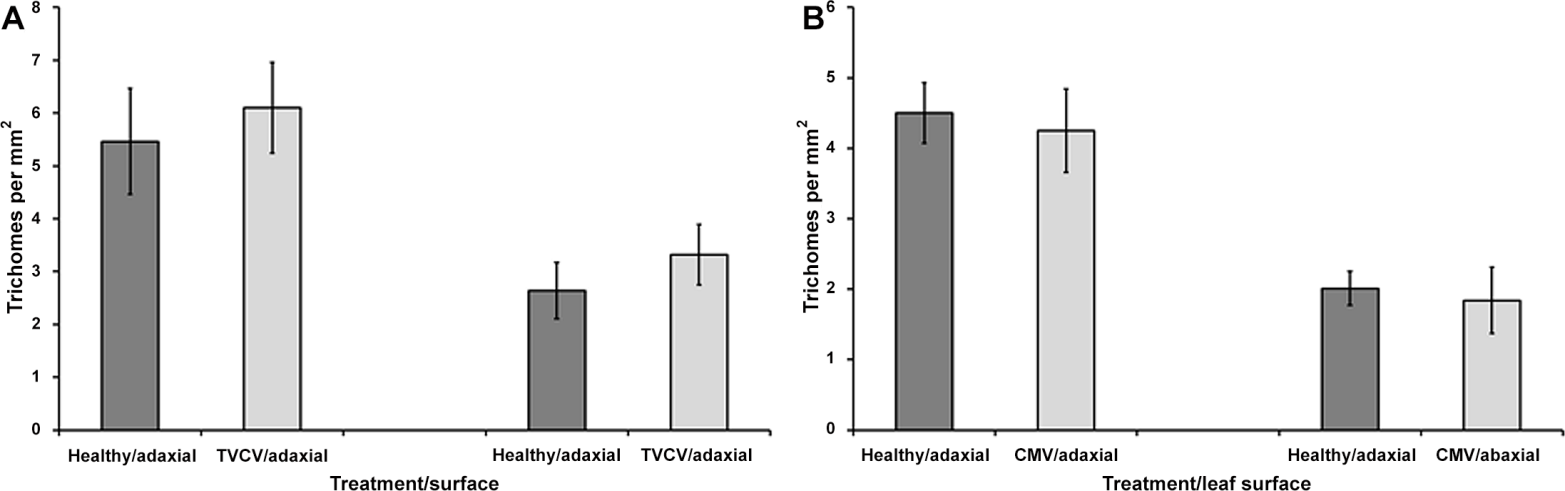
Average trichome densities on the adaxial and abaxial surfaces of TVCV (A) or CMV (B) infected *A*. *thaliana* leaves, in comparison to healthy controls. Error bars denote 95% confidence intervals. A total of 24 TVCV-infected leaves and 24 CMV-infected leaves (from separate plants) were analysed at 21DPI and compared to equal numbers of healthy controls. There were no statistically significant differences between healthy and infected leaves.

The TVCV infection did not significantly affect the rosette leaf trichome density on the adaxial (independent samples t-test: t = -0.944, df = 46, *P* = 0.35) or abaxial (independent samples t-test: t = -1.693, df = 46, *P* = 0.1) surfaces (Fig 7A).

Similarly, the trichome densities of CMV-infected leaves and healthy leaves showed no significant differences on the adaxial (independent samples t-test: t = 0.662, df = 46, *P* = 0.511) or abaxial (independent samples t-test: t = 0.646, df = 46, *P* = 0.521) surfaces (Fig 7B).

## Discussion

There was a notable difference in the effect of infection with aphid-transmitted and non-insect-vectored viruses on the percentage polarization of light reflected from systemically infected *N*. *tabacum* leaves. Infections with PVY and CMV, both aphid-vectored viruses, resulted in a significantly reduced percentage polarization in light reflected from the abaxial surfaces, whereas the non-insect-vectored viruses TMV and PepMV did not affect the percentage polarization of the reflection. On the adaxial surfaces PVY-infected leaves were significantly less polarizing than healthy leaves, whereas the decrease on CMV-infected leaves was not statistically significant.

These observed effects of virus infection on polarization reflection from *N*. *tabacum* therefore could possibly be associated with the transmission strategy of the virus, tentatively suggesting that viruses may gain adaptive benefits through alterations to leaf polarization reflection. It is typically observed that infected plants are more attractive to vectoring insects [3], and polarization-sensitive visual systems are common among insects. This is a tentative suggestion at this stage, as polarization sensitivity has not been demonstrated in viral vectors such as aphids and whiteflies. However, the widespread prevalence of polarization-sensitivity in insects that, like aphids, possess apposition compound eyes, makes it possible that aphids are able to perceive polarized light. Even if this were not the case, highly polarizing leaf surfaces typically reflect a white (broadband) specular glare [21], masking any underlying colouration (which can be perceived by humans despite our lack of polarization sensitivity), raising the possibility that a leaf with a lower percentage polarization could be more attractive to an insect simply because the pigmentation is more apparent, irrespective of the presence of a polarization vision system in the animal [41]. However, the potential salience of such changes in polarization reflection to insects in natural environments is not known. Another possibility is that a reduced percentage polarization affects the behaviour of predators, parasitoids or competitors of the viral vector. The seven spot ladybird, for example, has polarization-sensitive UV, blue and green photoreceptors [42], so an aphid-vectored virus may enhance its own transmission likelihood by influencing polarization reflection in ways which deter this aphid predator.

The percentage polarization reflection from a leaf is greatly influenced by the characteristics of the leaf surface, although reflection of light from the leaf interior also contributes to the percentage polarization reflection [21,33]. However, the PVY and CMV-induced reductions in the percentage polarization reflection from *N*. *tabacum* were observed in both the blue and green channel light on the abaxial surfaces, suggesting that alterations to surface reflection primarily underlie these differences. This is because surface-reflected light does not interact with the leaf pigmentation, and so the polarized component of light resulting from surface reflection is essentially broadband, or “spectrally flat” [21,33]. Had there been notable differences in the effects of infection on polarization in different wavebands, it would have suggested that altered reflectance from the leaf interior was to some extent responsible for the reduced percentage polarization reflection. It is therefore conceivable that the aphid-vectored viruses may manipulate the leaf surface phenotype (including micro-topography and epicuticular waxes, as discussed below) in order to alter the suitability or perception of the host plant to an insect vector. PVY and CMV are both NPT viruses [43,44], and as such would likely benefit from a reduced colonisation or feeding time by the vector because NPT viruses require rapid transfer between hosts for successful transmission, due to the transient association of virus particles with the aphid mouthparts [1,9].

In contrast to CMV-infected *N*. *tabacum*, CMV-infected *A*. *thaliana* leaves were significantly more polarizing than healthy leaves on the abaxial surface. Furthermore, infection with TVCV, a non-insect-vectored virus, also increased the abaxial percentage polarization reflection (in blue channel light). Comparing these viruses, there were therefore no differences associated with virus transmission strategy in the effects of virus infection in *A*. *thaliana*. It may be the case that the effects of viruses on polarization reflection are adapted to particular hosts, as it has been suggested that the induction of similar symptoms across a variety of hosts could arise as non-adaptive by-products of the infection process [45]. The abaxial surfaces of *A*. *thaliana* rosette leaves are unlikely to act as a useful visual cue for insects as they are located at ground level, although the colonisation of vectoring insects may be affected by an altered surface structure because whiteflies and aphids preferentially feed from the lower surfaces of leaves. It was on the abaxial surfaces of infected *N*. *tabacum* and *A*. *thaliana* leaves that the significant alterations to polarization reflection were observed; possibly these infections change the surface structure in ways which influence vector behaviours after alighting on the leaf.

It is known that leaf surface features affect the behaviour of insects. The structure and chemical composition of the cuticular waxes can affect feeding behaviour and whether a host plant is recognised as a suitable host [17,46], with aphids determining host suitability by making a series of short test punctures prior to engaging in sustained phloem ingestion [47]. NPT viruses would be likely to increase the likelihood of their transmission by causing symptoms which reduce the likelihood of sustained aphid feeding being initiated [2,3,6]; perhaps by changing the gustatory or olfactory properties of the waxy cuticle, or thickening the cuticle to make feeding more difficult. The epicuticular waxes also influence the polarizing properties of plants as they form the outermost layer of the leaf surface, and therefore represent the initial point of contact between incident light and the leaf. At a given angle of incidence, specular reflectance is influenced by the refractive index (RI) of a surface [48,49]. The surface wax RI, usually given as 1.5 for an air-leaf interface [50], could be affected by the composition of the wax layer [21] and therefore altered expression of wax synthesis genes during viral infection could lead to changes in the percentage polarization of reflected light.

Analysis of *CER* gene transcript levels suggested that PVY and CMV can affect expression of these genes in a contrasting way to TMV and PepMV. In *N*. *tabacum*, *CER6* showed a trend towards increased expression levels in PVY and CMV-infected leaves and reduced levels in TMV and PepMV-infected leaves. *CER6* also showed an increased abundance in CMV-infected *A*. *thaliana* leaves, albeit with reasonably high variance. *CER6* encodes a condensing enzyme, which extends VLCFAs greater than 24 carbons in length [35], so there may be a shift towards longer chain lengths or increased wax deposition on leaves infected with PVY/CMV in comparison to healthy or TMV/PepMV-infected leaves.

The only *CER* gene showing any trend towards notably increased abundance in leaves infected with a non-insect-vectored virus was the *CER9* gene in TVCV-infected *A*. *thaliana* leaves. *CER9* is a negative regulator of leaf wax synthesis [40], so when taken together with the absence of any increases in expression of the other *CER* genes, it appears that this non-insect-vectored virus may have inhibitory effects on cuticular wax synthesis relative to the impacts of the aphid-vectored viruses, where several examples of trends towards *CER* gene upregulation were observed across both host plants.

The experiments presented here were performed using *N*. *tabacum* and *A*. *thaliana* as viral hosts, both of which produce smooth, rather than crystalline, epicuticular waxes on the leaves. Any impact of altered wax gene expression on polarization reflection could be more pronounced on host plants with crystalline wax layers, as these structures are determined by the wax composition [51], meaning the surface roughness (and thereby polarization reflection) could be altered significantly through changes to the formation of epicuticular wax crystals.

Another feature of the leaf surface which could affect polarization reflection and vector behaviour is the trichome. A densely pubescent leaf will have a low percentage polarization reflection [19] and could interfere with insect movement [18], potentially causing aphids to abandon the leaf sooner than they would on a more glabrous leaf surface. Although there was not found to be any significant impacts of TVCV or CMV on trichome densities on *A*. *thaliana* leaves, it remains a possibility that viral infection may affect trichome numbers in other host plants, including *N*. *tabacum*. The absence of significant impacts of infection on leaf hair density in *A*. *thaliana* suggests that changes to the cuticular waxes may be primarily responsible for the altered polarization reflection, although general distortion to the leaf surface on a larger scale also has an effect on the percentage polarization reflection.

The observed impacts of the aphid-vectored viruses on polarization reflection in *N*. *tabacum* may therefore be a consequence of adaptive changes to the leaf surface structure induced by the viruses to influence vector behaviours after alighting on the leaf surface. It is also possible that the changes to polarization reflection influence host plant selection by vectors through visual detection of the variations in polarized reflectance between healthy and infected plants, changing their behaviour before they alight on a plant’s surface. Our observation in *N*. *tabacum* that aphid-vectored viruses can affect the percentage polarization reflection in a different way to non-insect-vectored viruses indicates that these effects are associated with transmission and are therefore likely to be adaptive, as the transmission stage is a major bottleneck in the viral lifecycle [52,53] and as such must be subject to very strong selection pressures. This suggestion is further supported by the fact that it is on the abaxial surfaces of the leaf that these effects are most strongly pronounced, as viral vectors such as aphids preferentially colonise and feed on the abaxial side of the leaf [47]. The abaxial surface seems the more likely to be used as a visual cue for apterous (wingless) aphids, which move between plants if host quality declines [54] and can spread viruses between closely spaced plants [55]; however, alate (winged) aphids, which can spread viruses over larger distances, would view leaves from a variety of angles, so the characteristics of both the adaxial and abaxial surfaces may influence the behaviour of alates.

The results of this study tentatively support the hypothesis that plant viruses affect the features of leaf surfaces in ways that are associated with the viral transmission strategy, specifically by altering the polarization of reflected light from the plant leaves reflect. Key areas to address in future investigations will be to determine whether aphid-vectored viruses also alter the chemical and/or physical properties of the leaf cuticle and whether this could impact aphid behaviour in ways which influence viral transmission, either through direct effects of the surface phenotype, or through remote cues mediated by the polarization of light. It would also be necessary to establish whether aphid-vectored and non-insect-vectored viruses differ in their impacts on polarization reflection in a variety of host plants, and to analyse in greater depth the impact of infection on the expression of the many genes known to regulate the formation of leaf surface structures. Finally, work specific to the polarization vision of aphids and the saliency to these animals of polarization reflections by plants is needed.

## Methods

### Polarization imaging

Images were acquired using a Nikon D70 SLR camera mounted on a tripod, with a linearly polarizing filter attached in front of the lens. An LED torch with diffusing material attached was used to provide depolarized illumination. Leaves were detached from plants and placed on a bench, in order to minimise movement whilst acquiring images, and the light source and camera were positioned at 55^◦^ from vertical, on opposite sides of the midline of the leaf, to obtain images with light reflecting from the leaf around Brewster’s angle. A series of seven images was taken per leaf, with the polarizing filter being rotated in 20° increments between each image, followed by a final “dark” image with the lens cap attached. Images were obtained in “raw” NEF format and converted to TIFF files using the open source software DCRAW. The relative brightness recorded at each pixel (after subtraction of the “dark” image) at various angles of polarizing filter orientation can be used to calculate the percentage polarization, and this was carried out using two custom written MATLAB programs. Data obtained in the blue channel, and the average of the two green channels (with peak sensitivities at 480nm and 540nm respectively) [56], were used for subsequent analysis. The average percentage polarization across a leaf was calculated from each image, using ImageJ to select all of the pixels in one half of the leaf lateral to the mid-vein. These values were then exported to MATLAB for analysis. When performing imaging experiments in *A*. *thaliana*, leaves were overlaid with card and a localised region of 0.5cm^2^ was imaged through a window cut in the card.

### Viruses

PVY° and a European isolate of PepMV were obtained from the Food and Environment Research Agency, York, UK. CMV Fny and TVCV were kindly provided by Dr John Carr, University of Cambridge.

### Amplification of *ECERIFERUM* genes

Sequences in the NCBI *N*. *tabacum* EST database with high homology to characterised *A*. *thaliana* mRNA sequences were used to design primers for amplification of *CER5* and *CER6* from *N*. *tabacum*. A degenerate primer approach was used to isolate the *N*. *tabacum CER3* sequence. The sequence data were subsequently deposited in the NCBI database (accession numbers KT279573, KT279574, KT279575). Primers are outlined in Table 1 (*N*. *tabacum*) and Table 2 (*A*. *thaliana*).

**Table 1:** Primer sequences used for qPCR experiments on *N*. *tabacum*.

Gene (*N*. *tabacum*)	qPCR primer sequence (5’-3’) /degenerate primer sequence (5’3’) where required
*CER3* FW	GCGCAATGGCTCTCCCAAGCT/AAYGARGCNYTNAAYGGNGGN
*CER3* RV	GGCGGAACGCTCTTTGTCGACA/NGCNCCNACYTCRTGRTG
*CER5* FW	GGCTCGAGGAGCTTGGTGGA
*CER5* RV	TCCGGTGAGGAGTGAAACCGCA
*CER6* FW	CTCCGGTTACCTGCCGAGTCCC
*CER6* RV	GCAGGAGGCAAGCACGTTTCTTCTC
*EF1α* FW	TCTCCAGGAGGCACTCCCTGG
*EF1α* RV	TGATGATGACCTGGGAGGTGA
*PDF2* FW	AGGCTTGCAGCCGGTGAATGG
*PDF2* RV	TTGTCGCAGCCGACCTTCGC

**Table 2:** Primer sequences used for qPCR experiments on *A*. *thaliana*.

Gene (*A*. *thaliana*)	qPCR primer sequence (5’-3’)
*CER3* FW	TTGACCAAGCCCACATGCCCAAC
*CER3* RV	TGTAATGTCGGCGATGCATGCACC
*CER5* FW	GGCGTGGGAAGATTTGACGGTGG
*CER5* RV	CTTGCGAGTCTACCTGCGAGAGA
*CER6* FW	TAGCTGAGAGCGATGGTGTGGGAG
*CER6* RV	GATGGACGCGGCTAGAAGCGA
*CER7* FW	AGGCAACATTGTGGTGATGGACC
*CER7* RV	TTCACGCCTTCTTCCCCCGG
*CER8* FW	TAGACGGAAAGCCGTCGGTA
*CER8* RV	TGGTCCAACCTTCTCATCAACA
*CER9* FW	GTATCCGTGTGCCTGTAGCG
*CER9* RV	GATGCTTGCAAACCTCGCA
*SAND* FW	GTTGGGTCACACCAGATTTTG
*SAND* RV	GCTCCTTGCAAGAACACTTCA
*F-BOX* FW	GGCTGAGAGGTTCGAGTGTT
*F-BOX* RV	GGCTGTTGCATGACTGAAGA

RNA was extracted from *N*. *tabacum* leaves at 7DPI and *A*. *thaliana* rosette leaves at 14DPI, using the RNeasy kit (Qiagen). For qPCR experiments, leaf material from five separate plants within each treatment was pooled prior to performing the RNA extraction. DNase I (Fermentas) was used to remove genomic DNA contamination and the first strand cDNA synthesis kit (Fermentas) was used to reverse transcribe the extracted RNA. DreamTaq green DNA polymerase (Fermentas) was used to amplify expressed target gene sequences from the first stand cDNA. Amplified sequences were cloned in *E*. *coli* using the pJET1.2/blunt vector (Fermentas). The plasmids were then extracted using the Qiaprep spin miniprep kit (Qiagen) and sequenced (GATC biotech) using the pJET1.2/blunt sequencing primers (Fermentas). BLAST searches were used to verify the identity of the amplified sequences.

### Quantitative PCR

qPCR experiments were performed using Maxima SYBR green (Fermentas). In each host, two reference genes were used for normalisation of expression levels; *ELONGATION FACTOR 1α* and *PROTODERMAL FACTOR 2* in *N*. *tabacum*, and *SAND* and *F-BOX* in *A*. *thaliana*. First stand cDNA was prepared as described above. Primer dissociation curves were used to confirm the specificity of primer pairs to their intended targets, and three or four biological repeats (each comprising independently inoculated groups of plants) were included in all experiments. Results obtained using both reference genes were averaged across all biological repeats, and analysed using the comparative ΔΔCt method [57]. One sample t-tests were used for statistical comparisons between the infected and healthy treatments. Primers are outlined in Table 1 (*N*. *tabacum*) and Table 2 (*A*. *thaliana*).

### Plant inoculation and growth

*N*. *tabacum* were germinated on Leavington F2 compost and grown for 5–6 weeks under long day conditions (16:8 hours light:dark) prior to inoculation. *A*. *thaliana* seeds were germinated on Lehle medium at 20°C under short day conditions (8:16 hours light:dark) and grown for 14 days, prior to transfer to soil (Leavington F2 compost with added sand), for a further 14 days before inoculation.

*N*. *tabacum* SR1 and the Colombia-0 ecotype of *A*. *thaliana* were mechanically inoculated with viruses. Infected material was ground in deionised water and the resulting sap was manually rubbed onto the upper surfaces of leaves using carborundum powder as an abrasive. The inoculum was washed from the leaf after two minutes. Healthy control plants were mock-inoculated using sterile deionised water. Expanding leaves close to the apex were inoculated on *N*. *tabacum* and upper leaves in the rosette were inoculated on *A*. *thaliana*. Following inoculation, plants were maintained at 20°C, under long day conditions in the case of *N*. *tabacum*, and short day conditions for *A*. *thaliana*.

### Trichome counts

Rosette leaves were detached from plants at 21DPI and placed between two microscope slides. The leaves were imaged using a Nikon D700 DSLR-CMOS sensor camera mounted on a Nikon SMZ-2T stereomicroscope. The “Volocity Demo” software was used to count the total number of hairs visible on each leaf surface, and ImageJ was used to calculate leaf surface areas for trichome density calculations.

### Statistical analysis

All statistical analyses were carried out using SPSS statistics (IBM). In the polarization imaging analysis, independent samples t-tests, or Mann Whitney tests where data did not meet requirements for parametric statistics, were used to test the significance of differences between healthy and infected leaves. The Benjamini-Hochberg correction (using a false discovery rate of 0.05) was used to account for the use of multiple statistical tests.
